# In Situ X-ray Absorption Spectroscopy Studies of Nanoscale Electrocatalysts

**DOI:** 10.1007/s40820-019-0277-x

**Published:** 2019-06-03

**Authors:** Maoyu Wang, Líney Árnadóttir, Zhichuan J. Xu, Zhenxing Feng

**Affiliations:** 1 School of Chemical, Biological, and Environmental Engineering, Oregon State University, Corvallis, OR 97331 USA; 2 School of Materials Science and Engineering, Nanyang Technological University, Singapore, 639798 Singapore

**Keywords:** X-ray absorption spectroscopy, Electrocatalyst, Nanoscale, In situ experiments

## Abstract

Nanoscale electrocatalysts have exhibited promising activity and stability, improving the kinetics of numerous electrochemical reactions in renewable energy systems such as electrolyzers, fuel cells, and metal-air batteries. Due to the size effect, nano particles with extreme small size have high surface areas, complicated morphology, and various surface terminations, which make them different from their bulk phases and often undergo restructuring during the reactions. These restructured materials are hard to probe by conventional ex-situ characterizations, thus leaving the true reaction centers and/or active sites difficult to determine. Nowadays, in situ techniques, particularly X-ray absorption spectroscopy (XAS), have become an important tool to obtain oxidation states, electronic structure, and local bonding environments, which are critical to investigate the electrocatalysts under real reaction conditions. In this review, we go over the basic principles of XAS and highlight recent applications of in situ XAS in studies of nanoscale electrocatalysts.

## Highlights

- This is the first review paper on the studies of electrocatalysts using advanced in situ X-ray absorption spectroscopy (XAS).
- This paper reviews the literatures to-date on new applications of in situ XAS (e.g., single-atom catalysts, surface reactions, nanoparticle size, and site occupation) that traditional XAS has not touched.
- This review focuses mostly on recent publications after 2010.

## Graphical abstract

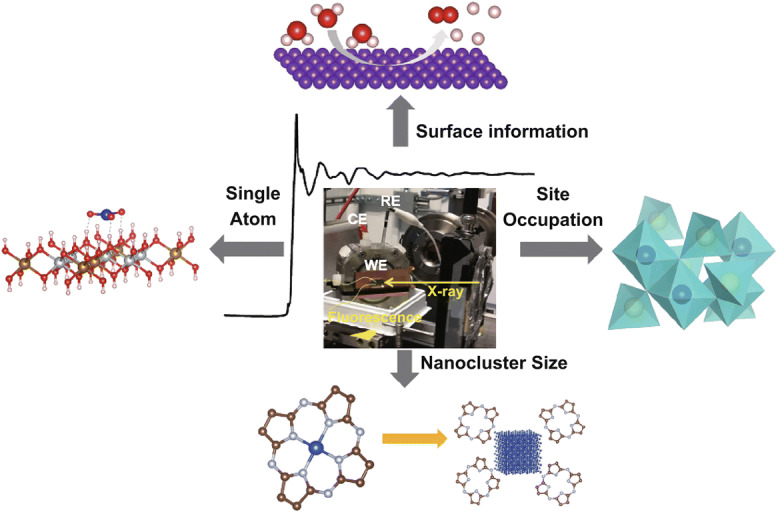

## Introduction

In the past decade, the growing global demands for energy and increasing awareness of environment pollution have motivated researchers to develop renewable energy devices that can convert green energy (e.g., solar and wind) into electricity or store them in clean fuels such as hydrogen [1–3]. Among various energy conversion technologies, fuel cells are the most efficient and clean systems as they can generate electricity by consuming hydrogen (hydrogen oxidation reaction, HOR) and oxygen (oxygen reduction reaction, ORR) that can be electrochemically reduced from water (hydrogen evolution and oxygen evolution reactions, or HER and OER, respectively) [4–6]. In addition, electrochemical carbon dioxide reduction reaction (CO2RR) is merging as one of the most promising methods to reduce the environmental pollution, as it can not only lower the greenhouse gas level, but also produce useful hydrocarbon (e.g., CH_4_) for fuel cells and other energy processes [7–10]. However, all these electrochemical reactions experience severe sluggish reaction kinetics, so electrocatalysts are necessary to improve the efficiency. In recent years, nanoscale catalysts have shown great advantages to catalyze electrochemical reactions due to their high surface area, tunable morphology, and a large amount of active sites [11–14]. For example, Zhou et al. demonstrated that a hollow nanospheres with mesoporous N-doped carbon shells and well-dispersed Fe_3_O_4_ nanoparticles can exhibit much higher ORR catalytic activity and better electrochemical durability than commercial Pt/C [15]. Additionally, Gupta et al. reported a bifunctional FeCoNi alloy nanoparticles on nitrogen-doped graphene that can reach the same ORR on-set potential as commercial Pt/C and 65 mV more negative OER on-set potential than commercial Ir metal [16]. On the hydrogen reaction side, Li et al. developed a selective solvothermal synthesis of nano-MoS_2_, which shows superior electrocatalytic activity in HER compared to other MoS_2_ catalysts [17]. Efforts have also been made on the CO2RR, as Cyrille et al. found a molecular Fe catalysts could electrochemically reduce CO_2_ to CO with a CO faradaic yield above 90% at low overpotential (0.465 V) through 50 million turnovers [18].

Although nanoscale catalysts exhibit intriguing activity and selectivity, their reaction sites are not well characterized. Furthermore, these nanocatalysts may experience restructuring during the reactions; thus, the true active phases for promoting electrochemical reactions are different from the initial material. For example, it has been found that a Pt-cluster-based catalyst is oxidized to a mixture of Pt(0), Pt(II), and Pt(IV), which are more active than Pt(0) during ORR [19]. Furthermore, several cases have shown that copper or copper compounds undergo reversible reconstruction processes to form different types of nano-clusters that promote CO2RR with high efficiency and superior selectivity (to either CO or CH_4_) [20, 21]. Therefore, it is critical to understand the structure of these materials, and particularly how they evolve during electrochemical reactions to identify the real structure–property relationships. Advanced characterizations are necessary to obtain such information, and in situ measurements are important to capture the structures that only exist in intermediate reaction states to understand the reaction processes. In situ X-ray absorption spectroscopy (XAS) has become a powerful technique to obtain oxidization states, electronic structure, and local coordination environment under reaction conditions. It can characterize many different materials including liquid and solid in either crystalline or amorphous structure, from bulk, to nanoscale and even a single atom [22–24]. In this review, we will go through the basic principles of XAS emphasizing the recent development of in situ XAS with applications in nanoscale electrocatalysis as well as further advancement of XAS in emerging research fields.

## Fundamentals of XAS

**Fig. 1:**
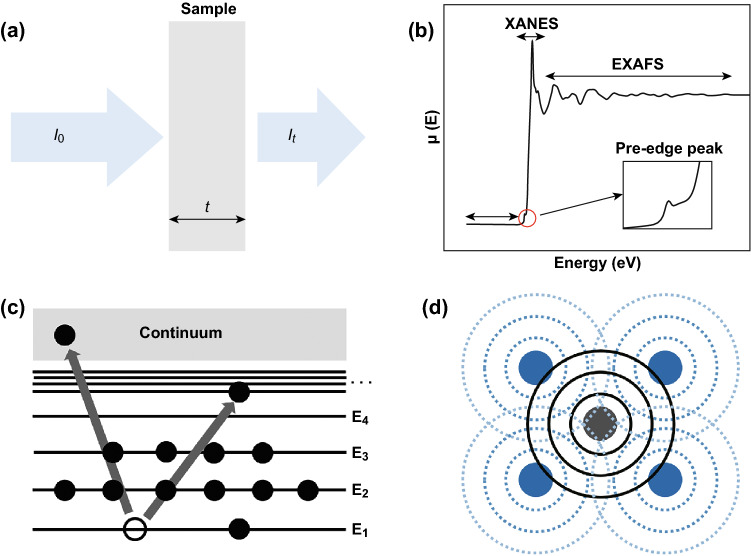
**a** Schematic of incident and transmitted X-ray beam. **b** Schematic of XAS including the pre-edge, XANES, and EXAFS regions. **c** Schematic of the X-ray absorption process and electron excited process, the black circle is electrons. **d** Schematic of interference pattern creating by the outgoing (solid black lines) and reflected (dashed blue lines) photoelectron waves between absorbing atom (gray) and its nearest atoms (purple). (Color figure online)

When X-ray passes through a material, the intensity is attenuated (Fig. 1a). According to Beer’s law, this attenuation can be characterized by the absorption coefficient according to Eq. 1,

1 $$I_{\text{t}} = I_{0} {\text{e}}^{ - \mu (E)t}$$

where *I*_0_ is the incident X-ray intensity, *I*_t_ is the transmitted X-ray intensity, *t* is the sample thickness, and (*E*) is the absorption coefficient that is dependent on the photon energy [22, 23, 25]. XAS measures the energy-dependent fine structure of the X-ray absorption coefficient [23–27]. When the incident X-ray energy is lower than the binding energy of the electron in the element’s orbital (say, *s* orbital), the electrons are not excited to highest unoccupied state or the vacuum. The lack of strong X-ray and electron interaction leads to the flat region shown in Fig. 1b; however, some unfavored transitions such as 1*s* to 3*d* in transition metals will appear as a pre-edge peak (Fig. 1b) [22–25]. Once the X-ray energies are high enough to excite core electrons to the unoccupied state (Fig. 1c), X-ray is strongly absorbed leading to a large jump in the spectrum, which is called the X-ray absorption near edge structure (XANES) (Fig. 1b). This region is sensitive to the oxidization state and electronic structure of the detected elements as the core electron energy is affected by the electron distribution in the valence state [22–25]. With the further increase in X-ray energies, the core electrons excited to continuum state (Fig. 1c) form the outgoing and scattering wave interference with neighboring atoms (Fig. 1d). The constructive or deconstructive interferences of the outgoing and scattering waves form the wiggles in the extended X-ray absorption fine structure (EXAFS) region (Fig. 1b), which reflects local atomic structure such as bond distance and coordination number [28–30].

**Fig. 2:**
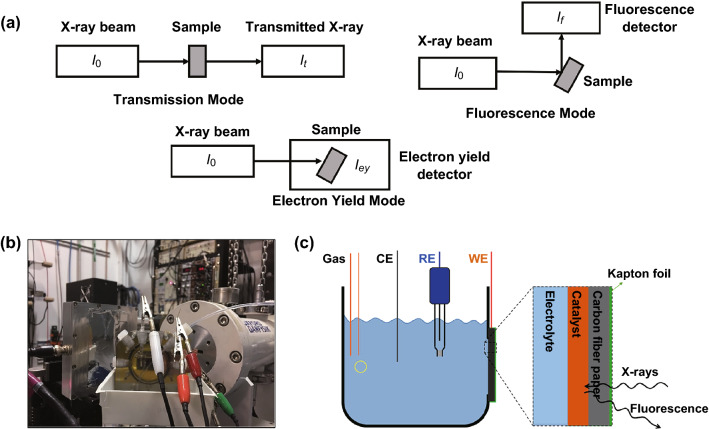
**a** Schematic of the experiment setup for three different XAS detection modes: transmission, fluorescence, and electron yield mode. **b** Photo of a real electrochemical cell for in situ XAS experiment setup. **c** Schematic structure of the electrochemical cell for in situ XAS setup experiments. Reprinted with permission from Ref. [21]

There are three basic modes for XAS signal collections, namely transmission, fluorescence, and electron yield modes (Fig. 2). Transmission mode measures the difference between incident and directly transmitted X-ray intensity (Fig. 1a). In transmission mode, concentrated and homogenous samples are recommended to increase the difference for high-quality data, in accordance with the Beer’s law [22–24]. In contrast, fluorescence mode measures the emitted X-rays from the elements (Fig. 2a). The intensity of this fluorescence is proportional to the absorption caused by the investigated element, but could also be affected by self-absorption effect. Thus, it is good for dilute, and works for, non-homogenous samples [22–24]. Instead of measuring the emitted fluorescence, we can also measure the emitted photoelectrons from the sample itself. Because of the relative short mean free path of photoelectrons, this mode is surface sensitive [22–24], while the two other modes are bulk sensitive. As XAS mainly probes the local structure, it can be used to measure many different materials including liquid and solid.

XAS requires synchrotron sources that can tune the X-ray energy easily. The fast development of synchrotron X-ray source (about 92 synchrotron sources around the world) has enabled the wide applications of XAS. Recently, XAS can also be performed on the benchtop instrument, making it more accessible in the laboratory [31, 32]. The use of hard X-ray (energy higher than 5 keV) can be beneficial for in situ measurements [21, 33, 34]. It is relatively hard to do in situ electrochemical reaction in many of the most advanced characterization tools such as scanning transmission electron microscopy, transmission electron microscopy, and X-ray photoelectron spectroscopy, which are frequently applied in the vacuum condition. In comparison, the continuous tunable hard X-ray running at the ambient condition, used in XAS, is much easier to operate in in situ experiment. The advanced electrochemical cell design also simplifies the operation of in situ XAS experiments. Figures 2b, c show a custom-designed XAS cell for electrochemical reactions in Feng’s group. It has four necks used for the gas inlet, and outlet, a reference electrode, and a counter electrode. The gas inlet and outlet make it possible to study reactions involving gases such as ORR and CO2RR. The front window is covered by the electrocatalyst as working electrode loaded on the carbon fiber paper, which is fixed by Kapton foil/tape to prevent any leakage of electrolyte. Combined with the reference and counter electrode, it is built as a three-electrode (or multi-electrode) reaction cell. Furthermore, the cell can hold up to 30 mL liquid that guarantees enough electrolyte for the electrochemical reactions. Different from laboratory-based electrochemical tests that involve a rotating disk to reduce the diffusion effect in the electrolyte, this XAS cell has been shown to serve as an excellent platform for in situ mechanistic studies [9, 21]. Currently, XAS is used not only to probe oxidization states and local structure but also to investigate nano-cluster size, element site occupation in the crystal lattice, and atomic disperse molecular structure due to the well-developed XAS techniques and analysis software.

## In Situ XAS Probing Oxidization State and Local Structure

**Fig. 3:**
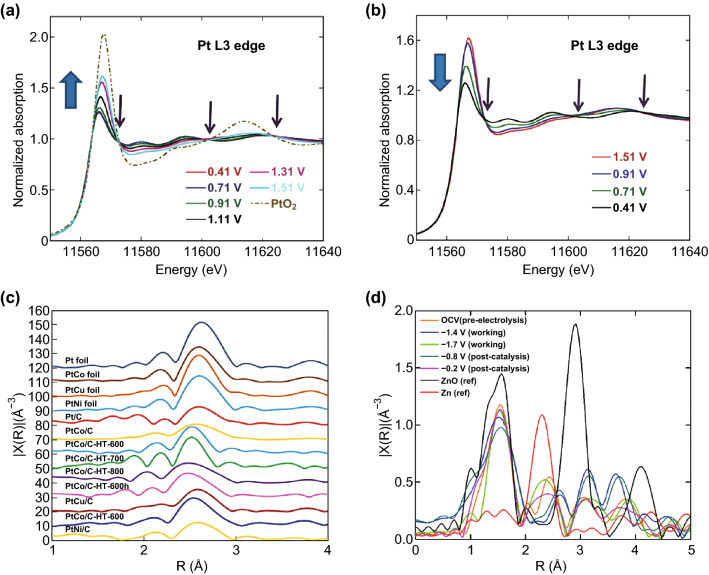
In situ XANES for Pt L_3_ edge of carbon-supported Pt nanoparticles at potentials **a** ascending from 0.41 to 1.51 V and **b** descending from 1.51 to 0.41 V in 1 M HClO_4_. Also shown (yellowish green dashed line) is ex situ XANES from commercial PtO_2_. Three distinct isosbestic points are observed at 11.573, 11.603, and 11.624 keV as designated by arrows. Reprinted with permission from Ref. [35]. **c** Fourier transform of k^3^-weighted Pt-L_III_ edge EXAFS oscillation of Pt foil, PtCo, PtCu, PtNi alloy foils, Pt/C, PtCo/C, PtCo/C-HTs, PtCu/C, PtCu/C-HT, and PtNi/C at 0.4 V versus RHE. Reprinted with permission from Ref. [38]. **d** Fourier transforms of Zn K-edge EXAFS spectra of the PorZn catalyst electrode at different potentials (V vs. SHE). ZnO and Zn are used as references. Reprinted with permission from Ref. [9]

Most electrocatalytic reactions involve chemical absorption and electron transfers, leading to the change of oxidization state and local structure, which is the short-range bonding structure, within 5Å, respectively [9, 21, 33–35]. These changes can be reversible and are difficult to detect by ex situ characterizations. XAS, particularly in situ, is capable of probing the oxidization states and local structure of selected elements under real reaction conditions. By using this technique, Sasaki et al. found a reversible oxidization state change of Pt monolayer electrocatalyst supported on Pd/C, which showed better ORR stability than commercial Pt/C [35]. The Pt monolayer was oxidized to Pt oxide with ascending potential (0.41–1.51 V), and Pt oxide was reduced back to Pt with descending potential (1.51–0.41 V), which is reflected by the change of the Pt L-edge XAS white line intensity (Fig. 3a, b). In addition, three distinct peaks at 11,573, 11,603, and 11,624 eV are observed in all the XANES spectra in Fig. 3a, b that indicates the coexistence of two different Pt species (Pt & PtO_2_). The percentage of the two Pt species is quantified by a linear combination analysis of XANES and suggests that the percentage of PtO_2_ formation per surface Pt atom on the Pt/C is almost 3.5 times higher than Pt monolayers. The formed PtO_2_ dissolves in the electrolyte, leading to decreased stability of the Pt/C. The formed and dissolved PtO_2_ reaction intermediate products are not detected by ex situ characterizations, and the difficulty in identifying this dissolvable intermediate, results in increased complexity in studying the catalyst stability. In contrast to this single-element nanocatalysts case, many materials have multiple elements such as alloy, metals supported on oxides, mixed metal oxides, and mixed metal hydroxyl, which are also widely used as electrocatalysts [36–38] but tracking each element’s change to determine whether catalysts have synergistically effect is very challenging. XAS, which is an element-specific technique, offers great advantages to study each element of the system separately; combined with in situ measurements, it provides the possibility to determine the role of multiple elements for catalytic reactions. For example, Wang et al. reported that the Fe-doped Ni(OH)_*x*_, with a high OER catalytic activity, forms Ni^3.6+^ and Fe^4+^ during electrochemical processes [33]. The Fe^4+^ leads to a strong covalent Fe–O bond, which interacts with Ni to form Ni–O-Fe bond, and the charge transfer between Ni and Fe through a “Ni–O-Fe” bond is the key reaction step, which results in high catalytic performance. These findings heavily rely on the analysis of in situ XANES measurements, which provide critical evidence for the synergistic effect of multiple metal elements as highly active nano-electrocatalysts.

Besides the oxidization states probed by XANES, the local coordination information provided by EXAFS can also be used to determine the active site of electrocatalysts or the role of multiple metal elements. Kaito et al. reported a core–shell Pt/Au electrocatalyst exhibiting higher ORR activity [39]. Their ex situ EXAFS studies found a shorter Pt–Pt bond in the Pt/Au than that in the Pt/C or Pt foil, but the shorter Pt–Pt was believed to contribute to the high ORR activity. Two years later, the same group, by using in situ XAS, identified the Pt–Pt bond distance as the primarily descriptor for ORR activity regardless of the atomic ordering or morphology of the different Pt nano-alloy [38]. A clearly linear trend was discovered increasing the ORR area specific activity with the decrease in the Pt–Pt bond (Fig. 3c). For instance, Pt_2_Co (PtCo/C-HT-600), which has the shortest Pt–Pt bond distance of the metals studied, exhibits the highest ORR activity which is approximately ten times more active per unit surface area than the commercial Pt/C at the reaction condition of 0.4 V versus RHE (Fig. 3c). Different from the synergistic effect of Fe-doped Ni(OH)_*x*_, here, the Co, Cu, and Ni in the Pt alloy are used to restrain the Pt–Pt bond, and the Pt itself is the active site for the catalytic reaction. It is common that catalysts metal centers play a major role for catalyzing reactions; however, there are some catalytic materials with metal as a non-active center [9, 40]. A Zn–porphyrin has been reported as a high activity electrocatalyst to convert CO_2_ to CO with Zn as a redox-innocent metal center [9]. In situ XAS shows no obvious change in Zn oxidization state, but variations in Zn EXAFS are found, which may be caused by the reduction in the porphyrin ligand or binding of molecules on the Zn site (Fig. 3d). Here, the porphyrin ligand, instead of the metal, works as a redox center for the CO_2_RR.

**Fig. 4:**
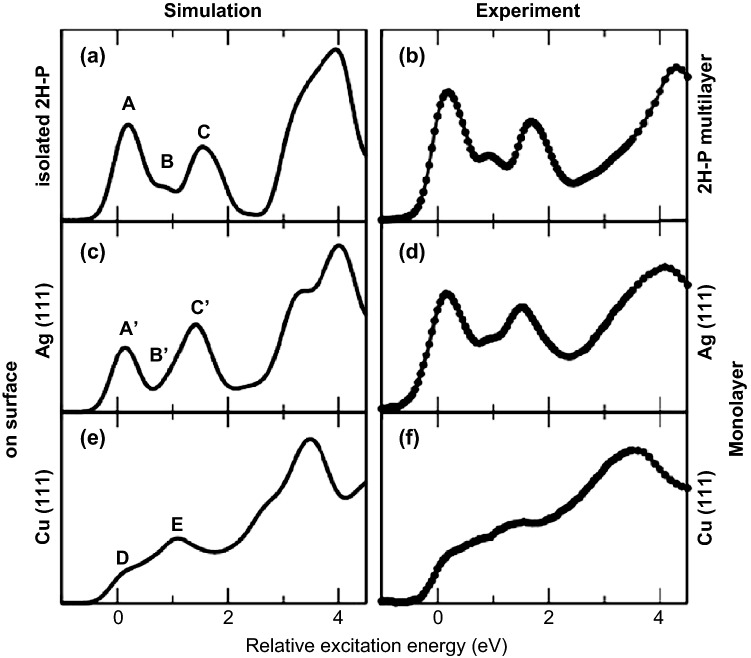
Comparison of experimental and simulated C K-edge NEXAFS spectra for 2-H porphine adsorbed on Ag(111) and Cu(111). Reprinted with permission from Ref. [61]

Many mechanistic studies of heterogeneous catalysts are complimented by density functional theory (DFT) calculation of reaction mechanisms, active sites, and adsorbate interactions. Many of these studies have used an indirect comparison between the XAS analysis and DFT calculations (e.g., activation energies at various reaction coordinates) to confirm active sites and favorable reaction paths, such as role of different active sites in HER activity of different CoP-based catalysts [41, 42], modified IrO_2_ and nanoscale SrRuO_3_ catalyst for OER [43, 44], zeolite chemistry under reactive SCR conditions [45, 46], and many more [47–50]. Coupling of DFT calculations with XAS simulations [51] and implementation of different spectroscopy modeling modules into common computational tools for materials modeling have made direct theoretical predictions and analysis of XAS spectra more accessible and provided molecular insights into catalytic reaction mechanism and active sites. Some of the most commonly used computer codes to calculate XAS spectra are FEFF, an ab initio code, developed by John Rehr, which is widely used in the field for multiple scattering calculations of X-ray spectra [26, 30, 52]. Other common computational tools are XSpectra, a post-processing tool, implemented into Quantum Expresso [53–55], an open-source electronic-structure calculations code, and CASTEP which recently added a core-level spectroscopy tool, but CASTEP is commonly used for materials modeling based on first-principles [56]. Using this combined approach, researchers have studied zeolites under reactive conditions and showed how H_2_O and NH_3_ adsorptions can be distinguished through the different Cu–O and Cu–N valence to core X-ray emission lines [45]. Theoretical XANES spectra for adsorbates on metal surfaces, based on DFT calculations, have shown general good agreement between experiments and theory [57–59]. For example, Diller et al. used dispersion-corrected DFT with transition potential approach [60] to compare carbon K-edge XANES spectra for porphine on Ag(111) and Cu(111) and found a very good agreement between experiments and theory with moderate computational effort (Fig. 4) [61]. XAS studies of reactions on surfaces have similarly benefitted from the combination of XAS- and DFT-predicted XAS spectra. A recent illustration of this is given by Saveleva et al. who identified the formation of an electrophilic oxygen species during OER on iridium oxides based on DFT calculations of the O K-edge XAS peaks [62].

## Probing Site Occupation

**Fig. 5:**
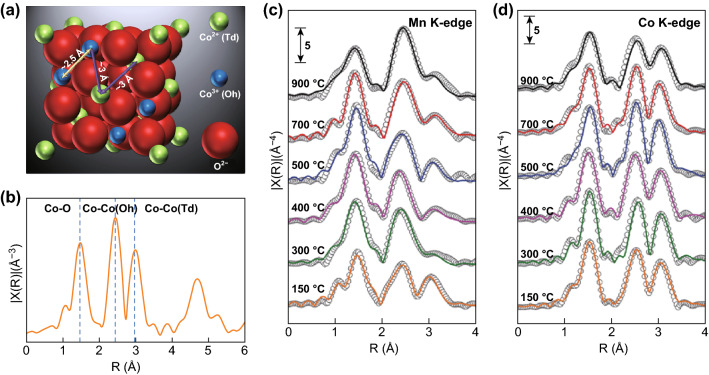
**a** The relation of interatomic distance between atom (Oh) and atom (Td) in spinel structure. **b** Co K-edge EXAFS spectra for Co_3_O_4_, the interatomic distances are shorter than the actual values owing to the fact that Fourier transform (FT) spectra were not phase-corrected. Reprinted with permission from Ref. [63]. EXAFS k^3^χ(R) spectra (gray circles) and fitting results (solid lines) of MnCo_2_O_4_ at **c** Mn and **d** Co K-edge. Reprinted with permission from Ref. [3]

Compared to precious metals, nanoscale metal oxides such as perovskite and spinel are becoming more and more popular as electrocatalysts as they contain earth-abundant and cost-effective elements [2, 6, 12, 36]. These oxides can have complicated crystal structures and various polymorphs, and the elements may occupy multiple crystal sites [3, 37, 63–65]. The crystal structure is a bulk property that can be studied by X-ray diffraction (XRD), but the site occupation requires local or short-range information that is hard to be probed by XRD. The distribution of elements at different sites, however, could influence the catalytic performance and thus is a critical parameter to obtain [3, 37, 63, 65]. A notable example is spinel oxide. Atoms in spinel octahedral (Oh) site have relatively short bonding distance compared to atoms in the tetrahedral (Td) site, as shown in Fig. 5a. This difference can be distinguished by EXAFS, as shown in Fig. 5b, and the site occupation can then be further quantified by model-based structure refinement. Wang et al. synthesized metal oxide Co_3_O_4_ in spinel structure, which shows reasonable OER activity [63]. The Co occupies both Td site and Oh site, but only the Co^2+^ in tetrahedral site plays an active role in the catalytic reaction. This conclusion was reached by using EXAFS that determines the site occupation of Co (Fig. 5b). Co in the Oh site has shorter Co–Co scattering path (~ 2.4 Å) than Co in Td site (3 Å), and those scattering paths in Fourier-transformed EXAFS are not phase-corrected. In situ XAS shows the local structure change of Co_3_O_4_ and suggests the formation of CoOOH intermediate species during the reaction. By comparing with the controlled samples of ZnCo_2_O_4_ and AlCo_2_O_4_, in which the Co^3+^ in Oh site is replaced by Zn and Al, respectively, the CoOOH is formed on the Td site, and is determined to be an active site for OER instead of Co^2+^ itself.

However, this study does not provide the Co distribution in Td and Oh sites, and the formation of CoOOH as intermediate product is based on the previous study. In a more recent study, Wei et al. performed a systematic investigation of different nanoscale transition metal spinel materials and utilized EXAFS together with model-based analysis to calculate site occupation of Mn atoms in Td and Oh sites [3]. By tuning the synthesis temperatures, the amount of Mn in the Oh site can be modified. The model-based analysis of ex situ XAS not only demonstrates the characteristic Oh peak (around 2.5 Å) and Td peak (around 3 Å) as shown in Fig. 5c, d, but also estimates the amount of Mn occupation in the two sites. By using the linear combination method to analyze XANES, the Mn valence state can be accurately determined. Subsequently the electron occupancy can be quantified using the known spin state. By correlating this with the electrochemical performance, the number of, e.g., occupancy for Mn in the Oh site is suggested as activity descriptor for both ORR and OER. This conclusion relies on the co-refinements of Co and Mn EXAFS, which ensures the accuracy for the estimation of atom occupation at Td and Oh sites.

## Probing Nanoparticle Size and Shape

**Fig. 6:**
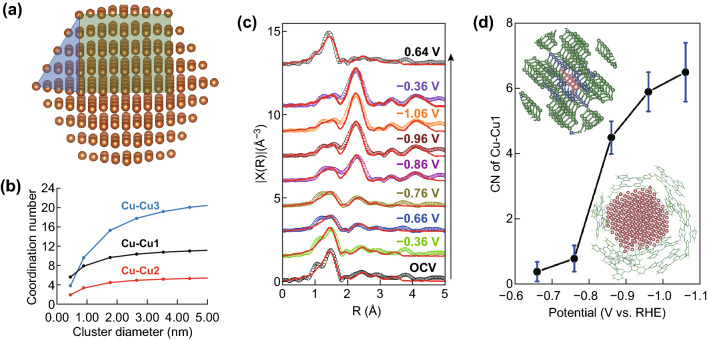
Cu nanocrystal model and its size-dependent Cu–Cu CNs for the first three shells of nearest neighbors. **a** A five-shell cuboctahedral Cu nanocrystal model. **b** Size-dependent Cu–Cu CNs for the first three shells of nearest neighbors for a cuboctahedral Cu nanocrystal. **c** Fitting results of the EXAFS spectra of the CuPc catalyst at different potentials in CO_2_-saturated 0.5 M aqueous KHCO_3_. Fitted R-space. **d** First-shell Cu–Cu CNs of the CuPc catalyst at different potentials. The upper left inset shows the CuPc crystal structure, and the lower right inset illustrates a possible configuration of the Cu nano-clusters generated under the electrocatalytic conditions. Color key: green-C; blue-N; pink-Cu. Error bars represent the uncertainty of CN determination from the EXAFS analysis. Reprinted with permission from Ref. [21]. (Color figure online)

The model-based EXAFS analysis can be further used to estimate the mean size and shape of nano-metal particles [66–68], providing important information to understand electrocatalysts’ restructuring in electrochemical reactions. The outer shells of nanoparticles are under-coordinated, thus reducing the total average coordination number measured by EXAFS. The nearest neighbors’ coordination number in nanoparticles has a nonlinear relation with the particle size diameter if the diameter is smaller than 3–5 nm [21, 68]. Figure 6b shows the coordination numbers of the first three shells of nearest neighbors for a cuboctahedral Cu nanocrystal as a function of the crystal size. This relationship is also highly dependent on the particle shape as the particle shape will influence the coordination number calculations. Many, extremely small, catalyst clusters are catalytic active [67], but EXAFS has become a premier method to investigate such small nano-cluster in electrochemical reactions. In many cases, the in situ XAS data could be nosier than ex situ ones due to the shorter data collection time, which makes the model-based fitting of EXAFS not accurate enough to determine the coordination number. In addition, the fitted coordination number has a common uncertainty around 1, thus adding additional error in particle size determination. Therefore, it is recommended to combine the model-based fitting with other characterization tools or theoretical calculation to predict the shape and size of nano-cluster. Due to the complexity of quantitative EXAFS analysis and particle size estimation, this application has not been widely used in electrocatalysis. A recent study reported a coexistence of square plan Fe^2+^-N_4_ and Fe/Fe_*x*_O_*y*_ nanoparticles as an ORR descriptor for metal-nitrogen-coordinated non-precious-metal electrocatalyst systems [68]. Their model-based in situ EXAFS analysis reveals the change of Fe–Fe and Fe–N coordination during the reaction, and the appearance of under-coordinated Fe–Fe bonding which is indicative of the formation of Fe nano-clusters. By referring to the electrochemical performance, Fe–N is the active site for both 2e^−^ transfer reaction to form HO_2_^−^ and 2e^−^ transfer reaction from HO_2_^−^ to HO^−^ in alkaline media. In contrast, in acidic media, the Fe–N is the primary active site for HO_2_^−^ formation, and Fe nanoparticles are the secondary active site for HO^−^. Although the authors did not report the nanoparticle size, the use of in situ EXAFS as well as model-based analysis plays an important role in determining the catalytically active sites and their corresponding role in the reaction.

Besides providing information related to the nanoparticle size, XAS is also useful to determine the shape of local coordination or nano-cluster formation during the reaction [33, 47, 69–71]. By analyzing the detailed coordination number of the nearest neighbors, one can construct the shape of the local building block. This is helpful when analyzing complex oxides that usually have both corner-sharing and edge-sharing unit cell. Bediako et al. reported that a nickel-borate with high OER activity restructured to an edge-sharing NiO_6_ octahedra during anodization process [69]. They found that Ni–B_i_ films exhibit a greater than two orders of magnitude increase in OER activity after catalyst anodization, which is caused by the formation of active Ni(III/IV) as probed by XANES. The model-based EXAFS analysis revealed an edge-sharing NiO_6_ octahedra with the domain size no smaller than 2 nm as the active sites. The analysis also suggested the short-range phase transition from β-NiOOH like Ni-B_i_ films to γ-NiOOH, and the γ-NiOOH may be intrinsically more OER active than β-NiOOH. Similarly, Kanan et al. reported a cobalt phosphate with Co-oxo/hydroxo clusters with high OER activity [71]; the quantitative EXAFS analysis suggests that the edge-sharing CoO_6_ octahedra of Co-oxo/hydroxo is the OER active site.

Frenkel et al., has summarized and reviewed the methods to model the structure and compositions of nanoparticles by EXAFS, which make the estimation of cluster size and shape easier [67]. Using Frenkel’s strategy, Weng et al. reported that a copper(II) phthalocyanine exhibits a high methane selectivity and yield for CO2RR, which is due to copper nano-cluster formation during the catalytic reaction [21]. In their work, the in situ XAS suggests a reversible oxidization state change of Cu center by XANES: Cu(II) is reduced to Cu(0) at the lowest applied potential and oxidized back from Cu(0) to Cu(II) when the potential is returned to the starting value, namely open circle voltage. Concurrently, the EXAFS also finds a reversible local structure change. The qualitative analysis of EXAFS demonstrates a descending trend of Cu–N coordination (corresponding to 1.5 Å Cu–N bond) and an ascending trend of Cu–Cu coordination (referring to 2.2 Å Cu–Cu bond) when the applied potential decreases to the reaction potential with the highest methane selectivity and yield (Fig. 6c, d), while an opposite trend is observed when the potential goes back to open circle potential. With the coordination number of Cu–N and Cu–Cu at each applied potential and by using a cuboctahedral metallic copper model (Fig. 6a), the authors conclude that the formation of 2-nm metallic copper nanoparticles under the reaction conditions is the true active site. This conclusion is supported further by density functional theory (DFT) which confirms that 2 nm is smaller than the nucleation size requirement (14.2 nm) and ensures the reversible structure change.

## Probing Atomically Dispersed Catalyst Structure

**Fig. 7:**
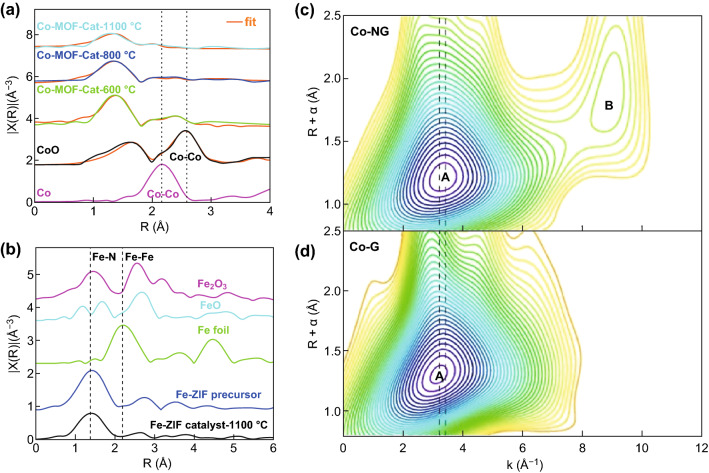
**a** Co K-edge EXAFS data and fits (orange). Reprinted with permission from Ref. [73]. **b** Fe K-edge EXAFS spectra for chemically Fe-doped ZIF before and after thermal activation. Reprinted with permission from Ref. [74]. Wavelet transforms for the **c** Co–NG and **d** Co–G. The location of the maximum A shifts from 3.2 Å^−1^ for Co–G to 3.4 Å^−1^ for Co–NG, indicating the presence of Co–N bonding in Co–NG. The vertical dashed lines are provided to guide the eye. Reprinted with permission from Ref. [77]

The ultimate development of nanoscale catalysts is toward single-atom catalysts. Atomically dispersed catalysts have high surface to bulk ratio and have been demonstrated to be promising candidates for numerous electrochemical reactions [21, 72, 73]. However, those materials have extremely low mass loadings, and majority of them are amorphous. It is challenging for most techniques to characterize those atomically dispersed catalyst to determine their structure information and related restructuring in reactions. The fluorescence mode of XAS is well suited to study samples in diluted or low concentration. The resolution of EXAFS is sub-angstrom, making XAS a unique probe to study single-atom catalysts. Wang et al. reported a nitrogen-coordinated single cobalt atom catalyst with respectable ORR activity and stability in acidic media [73]. The first coordination shell (around 1.4 Å) was fitted with Co–N, which shows a square planer CoN_4_ local structure (Fig. 7a).

The CoN_4_, with long-range disordered structure, was only found on samples annealed at 1100 °C, but samples annealed at 600 and 800 °C showed a mixture of Co–N and Co–Co coordination, suggesting only the sample annealed at 1100 °C is possibly atomically dispersed catalyst. The existence of Co–Co coordination worsens the ORR performance, and the highly dispersed atomic CoN_4_ sites are the key to promote the high performance. In this study, XAS helped determine the local coordination environment around Co. However, it is hard to distinguish bonding with elements that are close in atomic numbers in XAS, such as N and O or Co and Ni, due to the similarity in scattering path lengths and scattering intensities. In another study, Zhang et al. demonstrated a nitrogen-coordination single iron atom catalyst with almost the same catalytic activity as Pt/C [74]. The general EXAFS comparison between Fe–N and Fe–O (Fig. 7b) suggests almost same bond distance and similar peak feature. They did EXAFS fitting on those materials by modeling both Fe–N and Fe–O scattering paths and proposed two possible sceneries, one with four coordinated Fe–N and the other with five coordinated Fe–O. With a help from soft XAS at the Fe L-edge, which confirms the Fe local tetrahedral structure environment, the authors conclude that FeN_4_ is a more reasonable structure which enhances the ORR activity.

Instead of relying on model-based analysis on one dimensional (1D) data (e.g., Fourier-transferred amplitude vs. *k*), recent development in EXAFS analysis can include both k- and R-space to generate a 2D view of the EXAFS local structure. This is called wavelet transform which replaces the infinitely expanded periodic oscillations in normal EXAFS Fourier transform by located wave trains for the integral transformation [75]. The method recovers the primary EXAFS signal without any loss of information, but the magnitude of wavelet transform provides a radial distance resolution as well as the resolution in the k-space, which helps distinguish the different light elements such as nitrogen and oxygen coordination [76]. An example is shown by Fei et al. who reported that atomic Co on nitrogen-doped graphene is highly active in aqueous media with very low overpotentials (30 mV) [77]. To confirm the local coordination of Co, the wavelet transform on EXAFS data of Co suggests the change of Co–C bond in Co–G to Co–N bond in Co–NG due to a little shift of peak A from 3.2 to 3.4 Å^−1^ (Fig. 7c, d). They also found the heavy atom scattering such as Co–O and Co–O shows in the greater *K*-range, which will be helpful to identify the different coordinations. Although the wavelet transform help the analysis, it is still advised to combine it with other characterizations to provide further support when dealing with bonding with light elements.

Using ex situ XAS, many studies on atomically dispersed transition-metal catalysts have suggested that four nitrogen-coordinated metal center is the active site for electrochemical reactions [72–74]. However, in situ XAS by Tylus et al. found the coexistence of FeN_4_ species and Fe/Fe_*x*_O_*y*_ nanoparticles at reaction conditions [72]. In addition, recent study has reported that the nitrogen-doped carbon could also be the active site [78]. Strickland et al. studied a carbon-embedded metal atom clusters without direct metal-nitrogen coordination, which also achieve higher ORR activity than Pt/C in acidic media [40]. The qualitative EXAFS analysis finds only Fe–C and Fe–Fe coordination in FePhen@MOF-ArNH_3_ (FePhen) but no Fe–N coordination. The in situ XAS studies demonstrated no change in both oxidization state and local structure, suggesting that FePhen is not directly involved in the ORR. Based on the XAS results, they believed that the N-doped carbon mainly enhance the catalytic activity. Clearly, at the topic of single-atom catalysts, there will be more interesting, exciting, or sometimes surprising discoveries.Probing Surface Information

## Probing Surface Informaiton

**Fig. 8:**
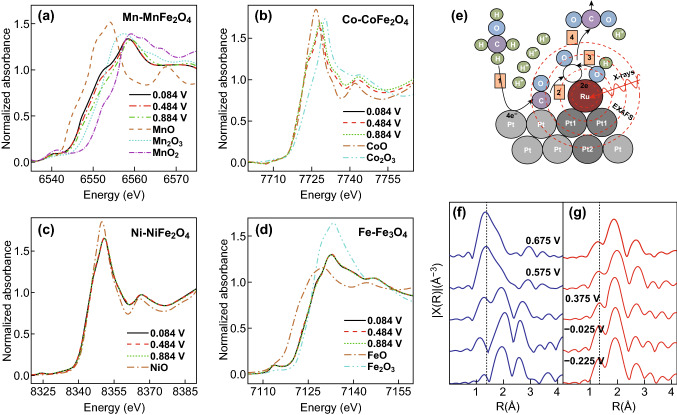
K-edge XANES spectra of **a** Mn in MnFe_2_O_4_, **b** Co in CoFe_2_O_4_, **c** Ni in NiFe_2_O_4_, and **d** Fe in Fe_3_O_4_ under various applied potentials. Reprinted with permission from Ref. [37]. **e** Schematic of the surface reaction of the Ru atoms. R-space graphs as a function of applied potential **f** without (blue) and **g** with (red) CH_3_OH. Dotted line placed at ca. 1.4 Å signifying a reference point for the oxygen peaks. Reprinted with permission from Ref. [82]. (Color figure online)

Most in situ XAS measurements rely on hard X-ray (energy higher than 5 keV) that is deeply penetrating, and thus not inherently surface sensitive [23, 24, 79]. However, electrocatalytic reactions occur on the surface of the materials while the XAS measures the bulk average, which limits the application of in situ XAS in the electrochemistry. Benefiting from the electron yield mode that collects electrons with short mean free path (~ 10 Å), XAS can be tuned to gain surface sensitive [80]. Wang et al. applied such strategy to characterize the Fe and Co surface electronic orbits for LaCo_*x*_Fe_1−*x*_O_3_ perovskite electrocatalysts in ORR to predict the descriptor for the reaction pathway [81]. Thus far, there is no advanced in situ cell designed to work in electron yield mode as the electron excited by X-ray could be interfered by applied voltage in electrochemical reaction. Few reports can be found for surface-sensitive hard XAS. Despite the difficulties, hard XAS can still be used to probe the surface oxidization state and local structure change for some specific cases, particularly when reaction occurs only on the surface. For instance, the catalytic reaction for pseudo-capacitive mainly takes place on the surface or the near-surface region [37]. When using nanoparticles with size smaller than 10 nm, the signal collected by XAS is actually dominated by surface contributions, making in situ XAS surface sensitive for the investigation of the pseudo-capacitance performance of spinel ferrite nanoparticles XFe_2_O_4_ (X = Mn, Fe, Co, Ni) [37]. For controlled parameters Fe, Wei et al. showed no oxidization state change during the charging process, and other elements such as Co and Ni also exhibit no detectable oxidation state change (Fig. 8a–d) [37]. However, the in situ XANES on Mn displays significant edge shift toward the higher energy as a function of the applied potential, and the corresponding EXAFS demonstrates that Mn occupying octahedral sites are responsive for the pseudo-capacitance. Due to the nano-size and surface reaction on spinel oxides, the information revealed by bulk-sensitive XAS becomes surface-sensitive due to the proportionally large contribution from the surface.

Furthermore, some other materials limit the reactions only to the surface [82–84]. Pelliccione et al. reported a PtRu catalyst for electrochemical oxidization of methanol on surface Ru atoms (Fig. 8e) [82]. The PtRu was synthesized by depositing Ru atoms only on the surface of Pt nanoparticles. Therefore, probing changes of Ru atoms turn a bulk-sensitive XAS into a surface technique. In this study, Ru gradually oxidizes from the metallic Ru to Ru(III)/Ru(IV) mixture at the highest potential in the background electrolyte, but in the presence of methanol, the Ru remains a mixture of Ru(0) and Ru(III). The model-based EXAFS analysis indicates the existence of Ru–O to form RuO_2_-type coordination during the reaction in the background electrolyte in good agreement with the XAS experiments. Interestingly, in situ EXAFS finds the adsorption of OH and CO on the Ru atoms, and the oxidization of CO with coadsorbed OH occurs faster than oxidization of Ru to higher state (Fig. 8f–g), suggesting that Ru atoms promote the catalytic reaction. Another similar study used a well-defined monolayer of Pt on single crystal Rh(111) substrate for ORR. In this case, all Pt signals from XAS come from the Pt surface atoms and is therefore surface-sensitive [85]. The authors observed an ascending trend of Pt oxidization state with increased applied potential, and the EXAFS indicates an increasing coordination of Pt-O and a decreasing coordination of Pt–Pt, which is consistent with the change of oxidization state. The direct observation of O adsorbing on the Pt/Rh(111) surface by in situ XAS also confirms the theoretical prediction. These examples demonstrate that the bulk-sensitive XAS can also work well in special cases to study the surface restructuring for nanoscale electrocatalysts.

## Other XAS Applications

Although this review mainly focuses on hard XAS for in situ studies, the tender and soft XAS with energy lower than 5 keV are also powerful tools to characterize detailed electronic structure such as the orbit spin and splitting for 3*d* transition metal, and the transition-metal–oxygen covalency [73, 74, 86]. For example, Suntivich et al. reported a method to estimate the hybridization between transition metal and oxygen by using the oxygen K-edge XAS, and Hocking et al. demonstrated the 3*d* orbit splitting in either octahedral or tetrahedral field with either high-spin or low-spin state by combining Fe L-edge XAS and theoretical simulation. In addition, soft XAS can probe bulk structure information such as crystalline or amorphous states and site occupation. For instance, Wu et al. distinguished amorphous and crystalline SiO_2_ through analysis of the peak features of the Si K-edge XAS by analyzing the peak features [87], and Zhang et al. demonstrated that the Fe L-edge XAS can be an indicator of tetrahedral and octahedral coordination [74].

Besides XAS working at different energies, there are two emerging types of XAS that provide temporal resolution, namely quick XAS and ultrafast XAS (or pump-and-probe spectroscopy). Due to lengthy time for data collection during XAS experiments, common in situ XAS measures the thermodynamic steady states. Quick XAS enables time-resolved XAS by using a dedicated X-ray monochromator to ensure rapid and repetitive energy scan, which results in measurements as short as couple of seconds [88]. Quick XAS can achieve 10-ms time resolution and can be applied to study dynamic or kinetic states in chemical and electrochemical reactions such as high rate delithiation behavior and nucleation process of nanoparticles [89, 90]. Currently, the quick X-ray absorption spectroscopy has also been used in the electrocatalysis field to resolve the reconstruction of the catalysts within several seconds [91, 92]. Pump-and-probe spectroscopy studies ultrafast electronic dynamics. In this technique, a stronger beam (pump) is used to excite the sample to generate a non-equilibrium state which exhibits the relaxation dynamics in the femtosecond or picosecond time regime, and a weaker beam (probe) is applied to observe the pump-induced changes in the optical constants [93, 94]. The function of changes in the optical constants with a time delay between pump-and-probe pluses describes the relaxation of electronic states in the sample. Therefore, ultrafast XAS can reach even higher time resolution (around femtosecond second) than quick XAS and can resolve intramolecular dynamics such as real-time observation of valence electron motion [95, 96].

## Conclusions

In summary, we reviewed the recent progress of in situ XAS applied in the nanoscale electrocatalysis and discussed some other margining applications of XAS. XAS is a useful technique for understanding the local coordination environment, electronic structure, and oxidization state of selected elements. Moreover, in situ XAS can probe the material local structure and valence state under real reaction conditions to determine active site for rationalizing the design of electrocatalysts. XAS has almost no special requirements on sample type or preparation and is being applied to investigate the atomically dispersed materials in low loading and extremely small nano-clusters under harsh electrochemical reaction conditions. The advanced analysis software enables more quantitative analysis of the EXAFS to extract useful structural information such as surface absorption and nano-cluster size and shape. In addition, the bulk-sensitive XAS can be used to probe the surface modification in some special cases such as for surface reaction or monolayer materials. With the development of new generation (e.g., 4th) synchrotron X-ray sources with higher flux, stronger coherence, and better time-resolution, XAS can be applied in merging fields to explore new frontiers in nanoscale electrocatalyst and related restructuring to facilitate the understanding of the structure–property relations for the design high-performance and low-cost electrocatalysts.
